# Genetic and morphological characterization of United States tea (*Camellia sinensis*): insights into crop history, breeding strategies, and regional adaptability

**DOI:** 10.3389/fpls.2023.1149682

**Published:** 2023-05-12

**Authors:** Caitlin Clarke, Brantlee Spakes Richter, Bala Rathinasabapathi

**Affiliations:** ^1^ Horticultural Sciences Department, University of Florida, Gainesville, FL, United States; ^2^ Plant Pathology Department, University of Florida, Gainesville, FL, United States

**Keywords:** InDel markers, Nei’s genetic distance, *Camellia sinensis*, tea yield, biohistory

## Abstract

Multiple introductions of tea (*Camellia sinensis*) to the United States since the 1850s have resulted in US tea germplasm that are currently poorly characterized. To resolve questions concerning the relatedness and regional adaptability of US tea germplasm, 32 domestic individuals were evaluated using 10 InDel markers, and compared with a background population of 30 named and registered Chinese varieties of tea. The marker data were analyzed *via* a neighbor-joining cladistic tree derived from Nei’s genetic distance, STRUCTURE, and Discriminant Analysis of Principal Components, which revealed four genetic groups. Nineteen individuals selected from the four groups were assessed for seven leaf traits, two floral descriptors, and leaf yield, to identify plants best adapted to Florida field conditions. Our analyses compared with available historical records led us to estimate the most likely provenance of some of the US individuals, to precisely identify tea plant material and to choose most diverse accessions for breeding tea improved for adaptability, yield and quality.

## 1 Introduction

Tea (*Camellia sinensis*) is one of the most-consumed beverages globally. Its popularity is due to both cultural precedent and documented health benefits (Chen and Lin, 2015). Despite a growing market for tea in the United States (US), almost all tea sold in the US is imported (FAO, 2018; Zhang et al., 2020). A few small (<100 acre) tea growing operations exist in the Southeast and Pacific Northwest regions, with only one extant large-scale (>100 acre) tea farm operating in coastal South Carolina. Currently, the market for loose leaf and specialty tea is growing (Goggi, 2022). At the same time, the number of small-scale tea farms in the Southeastern US, which would be more likely to supply this market than the commodity tea market, is growing as well (Price, 2021). The nursery stock for tea that is domestically available in the US has a complex history (D’Auria et al., 2022) which includes almost two hundred years of interbreeding among historical stocks and two introductions of new cohorts of germplasm in the past sixty years (Walcott, 1999). Available germplasm is not well-characterized, presenting difficulties both for farmers attempting to choose the best plants for their environment, and for plant breeders attempting to develop optimal breeding strategies.

Tea is an evergreen perennial that was likely first domesticated in southwestern China around 3000 BCE, according to historical evidence (Wambulwa et al., 2021). Tea prefers well-drained, acidic soils and temperatures between 12 and 30°C during the growing season (Ahmed and Stepp, 2013). Most tea is produced in humid tropical or humid subtropical climate regimes; however, different varieties appear to be adapted to local climatic conditions (Xia et al., 2020).

Since tea’s first domestication resulting in China-type teas, the crop has undergone possibly two more domestication events in southwestern China and Assam, India, resulting in Chinese Assam-types and Indian Assam-types (Meegahakumbura et al., 2016). The vast spread of tea in antiquity and long history of human cultivation have resulted in area-specific landraces and clonal varieties. These generally fall into one of two recognized subspecies, China-type, *Camellia sinensis* var. *sinensis* (CSS) and Assam-type, *Camellia sinensis* var. *assamica* (CSA). However, cross pollination followed by continuous selection has resulted in many varieties exhibiting varying levels of admixture between the two. Generally, CSS is characterized by small leaves and a spreading habit, while CSA has larger leaves and a semi-arboreal habit (Carr, 2018). However, identification based on morphological traits has limited utility because of the continuous nature of the characteristics (Wang et al., 2020).

Historical records reveal a complex past of tea cultivation in the US, particularly in the Southeast, and with regard to the provenance of plants. Junius Smith’s 1849 attempt at tea cultivation in South Carolina used seeds and plants sourced from India with some possible admixture of CSS plants from Guandong, China (Rose, 2010; Walcott, 2012). Much of the tea propagated in the eastern US in the 1850s and 1860s originated from Anhui, Fujian, and Zhejiang provinces in China, sourced by Robert Fortune for the Royal Horticultural Society (Gardener, 1971). The Pinehurst tea farm in South Carolina, established in the 1880s, included an Assam-Chinese hybrid that likely predates Fortune’s expedition; this plantation also included plants from Darjeeling, as well as an unnamed Japanese variety, and ‘Dragon’s Pool’ seed from Zhejiang (Walcott, 1999). The full extent of tea germplasm exchange from East to West cannot be quantified; however, given the federal support of tea cultivation, it is likely that in the 19^th^ century, tea germplasm was imported to the US from China, Japan and India (Klose, 1950). In the 1960s, Lipton installed experimental stations for tea production around the US Southeast and West. Though Lipton had largely abandoned US tea production by the 1980s, some of the tea lines they introduced are currently being revived. In addition to the historical varieties, more recent (<40 years) efforts to produce tea by nursery growers and hobbyists have led to new introductions from China, India, Russia, Georgia, Japan, Korea, and Nepal. These manifold geographical origins, together with up to 200 years of hybridization, and the inclusion of varied recent additions, complicates the questions of 1) selecting area-appropriate germplasm for growers and 2) optimizing breeding strategies for researchers.

Where pedigrees are lacking, investigating the genetics of tea plants is important for making decisions about controlled crosses in a breeding program. DNA-based methods of identification have been shown to produce adequate conclusions about ancestry, admixture, and phylogenetic relationships (Liu et al., 2017; Jin et al., 2022). Availability of the genome sequence for tea (Wei et al., 2018; An et al., 2020) facilitates the generation of DNA-based markers. Research performed on Chinese tea has shown that local varieties retain genetic signatures related to the region to which they are adapted (Liu et al., 2019). Supporting this, phylogenetic studies performed using plastid genomes (Wambulwa et al., 2017; Meegahakumbura et al., 2018), SSR markers (Tan et al., 2016; Liu et al., 2018), SNP and InDel markers (Liu et al., 2019) have found a strong correlation between clades and geographical origins of plants.

Given the limited nature of information regarding provenance and performance of US tea, this research was performed to characterize the genetic diversity and potential geographic origins of 32 tea accessions collected from sources in the southeastern US. Ten InDel markers developed by Liu et al. (2019) were used to estimate the genetic diversity of the tea accessions. The study included 30 of Liu et al. (2019)’s reported Chinese varieties as a background population, against which the US accessions were compared, thus deciphering geographic origin for some of the University of Florida tea accessions. Morphological descriptors and yield were analyzed for 19 of the accessions to test relationships between markers and yield phenotype.

## 2 Materials and methods

In previous studies from our research group (Orrock et al., 2017; Orrock et al., 2020; Orrock et al., 2021), we have used plant names provided by the nursery or other sources as names of “accessions.” However, recognizing extent of variability within commercially sourced plants sold under a single trade name, here we revise our terminology on germplasm to better reflect the diversity. “Named groups” are tea plants obtained with a trade name provided by the commercial source. Genetically unique individuals within a named group are considered “accessions.” Wherever the derivation of a named group (clonally or sexually propagated) is unclear, single plants will be referred to as “individuals.”

### 2.1 Tea plants

Thirty-two domestically sourced individuals of tea were included in this study (**Supplementary Table 1**). Nineteen individuals from eight named groups were sampled from the genetic diversity and yield study plots at Plant Science Research and Education Unit in Citra, FL (29°24’27N, 82°14’11W). Sampled individuals were chosen for the genetic evaluation based on annual yield totals for 2021 (**Table 1**). Within each named group, the highest-yielding and lowest-yielding individuals were included. For the three highest-yielding named groups, ‘Small Leaf,’ ‘Fairhope,’ and Miwa’s Garden,’ a median-yield individual was also sampled for the genetic diversity study. Seven named groups (‘Assamica,’ ‘Big Leaf,’ ‘China Seed,’ ‘Fairhope,’ ‘Georgian,’ ‘Large Leaf,’ and ‘Small Leaf’) were installed in 2016, and one (‘Miwa’s Garden’) was installed in 2020. The site is maintained with drip irrigation and weed barrier fabric, and plants are fertigated weekly with 6 lbs of N, 8.5 lbs of S, and 7 lbs of K per acre. The nineteen individuals representing eight named groups were also used for measuring morphological descriptors. Leaf samples from three individuals growing at the Great Mississippi Tea Company in Brookhaven, MS and an additional ten container-grown accessions maintained in a greenhouse in Gainesville, FL were included in the genetic diversity study (**Supplementary Table 1**). The greenhouse plants were used to determine timing of anthesis. Thirty tea accessions sourced from Anhui Agricultural University in Hefei, China were included in the genetic diversity study as a background population against which the unknown accessions were compared (**Supplementary Table 2**). Each of these accessions is a representative from a registered Chinese variety.

**Table 1:** The field-grown tea plants used in the genetic diversity study and their respective total annual yield (g/plant/year).

Named Group	High yield (g/plant/year)	Individual	Low yield (g/plant/year)	Individual	Median yield (g/plant/year)	Individual
‘Assamica’	22.3	Assamica 2	9.9	Assamica 1	NA	NA
‘Big Leaf’	91.1	Big Leaf 1	2.2	Big Leaf 2	NA	NA
‘China Seed’	27.7	China Seed 1	0.1	China Seed 2	NA	NA
‘Fairhope’	142.6	Fairhope 3	3.7	Fairhope 1	32.8	Fairhope 2
‘Georgian’	64.2	Georgian 2	4.7	Georgian 1	NA	NA
‘Large Leaf’	90.6	Large Leaf 2	3.4	Large Leaf 1	NA	NA
‘Small Leaf’	135.1	Small Leaf 2	5.1	Small Leaf 3	54.6	Small Leaf 1
‘Miwa’s Garden’	158.5	Miwa’s Garden 2	10.9	Miwa’s Garden 3	47.0	Miwa’s Garden 1

Within each named group, individuals were chosen to be representative of the highest and lowest yield from the mean of 2020 and 2021, marked as high yield and low yield respectively. ‘Miwa’s Garden’ idividuals were chosen from 2021 yield data, as this named group was not harvested in 2020. For the three highest-yielding accessions, individuals with a median yield is also included (NA, not applicable).

### 2.2 Yield

Yield was assessed over 3 harvests in 2020 and 4 harvests in 2021. Yield measurements included all plants within each of 8 named groups on the site. Plants were harvested by plucking the apical meristem and first two expanded leaves, commonly referred to as a “two and a bud” pluck. Yield in fresh weight was measured per-plant immediately after harvest to prevent loss of mass due to transpiration.

### 2.3 Morphological measurements and anthesis characterization

**Figure 1:**
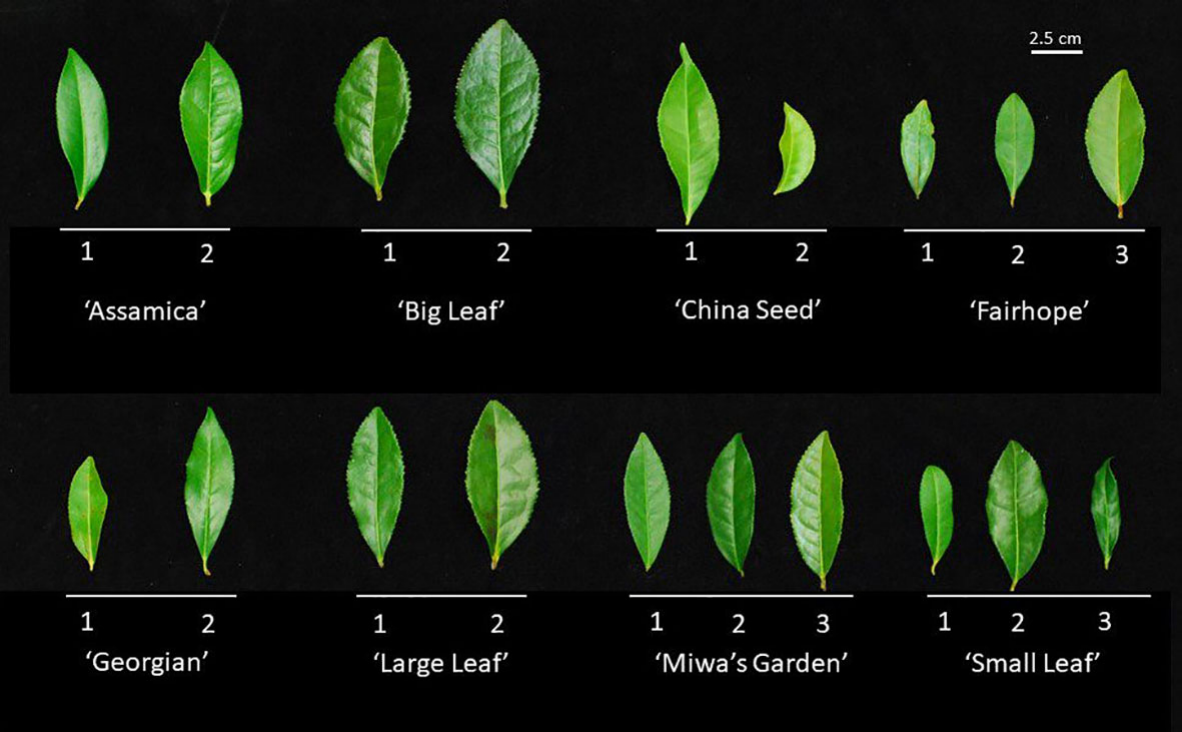
Representative first fully expanded leaf on mature wood from each of the 19 field accessions used for morphological characterization and genetic distance analysis. Leaves above a common bar belong to the same named group, labeled below. Numbers correspond to the alphanumeric designation used to identify the individual plant. Leaves were evaluated for length, width, length:width ratio, margin serration, and color.

Morphological descriptors were measured on field-grown plants to describe leaf and flower characteristics as outlined in the IPGRI descriptor tool (IPGRI, 1997). Seven leaf characters (Internode length, mature leaf color, leaf margin, leaf length, leaf width, leaf length, and leaf pose) and two floral characters (relative height between pistil and stamen, and style splitting) were measured using calipers and visual inspection, as appropriate, in January 2022. Mature leaf descriptors were recorded on the first fully expanded leaf occurring on mature wood (**Figure 1**). Anthesis was measured from February through April of 2021 on greenhouse plants using time-lapse photography. Photographs of the floral bud were taken in 10-minute increments over a 24–48-hour period, starting at balloon stage and ending after the completion of anthesis. Photographs were time-stamped to accurately determine beginning and end times of anthesis.

### 2.4 DNA extraction and amplification

Young leaves were sampled and frozen at -20°C until DNA extraction. Genomic DNA was extracted using the DNEasy Plant Kit (Qiagen) and quantified using NanoDrop (Thermo Fisher Scientific, Waltham, MA, USA). Concentration of genomic DNA was adjusted to 10 ng.µl^-1^ prior to PCR amplification. Fluorophore 6-FAM-tagged primers were used for PCR amplification (**Supplementary Table 3**). PCR amplifications were performed in 20 uL reactions each containing: 10 µL Plant Phire DNA polymerase master mix (Thermo Fisher Scientific), 1 µL forward primer (0.5 µmol), 1 µL reverse primer (0.5 µmol), 1 µL genomic DNA (10 to 20 ng), and 7 µL water. Cycling conditions were as follows: 5 minutes initial denaturation at 98°C; 35 cycles of 5 seconds denaturation at 98°C, 5 seconds annealing at primer-specific temperature, and 20 seconds extension at 72°C; and 5 minutes final extension at 72°C. Products were verified on 2% (wt/vol) agarose gel, and fragment size was determined using capillary electrophoresis (Applied Biosystems 3730 Genetic Analyzer, Waltham, MA, USA). Alleles were called in PeakScanner version 1.0. Missing alleles were coded as “0” for null alleles. Five fragments amplified from CsIndel17 were sequenced with Sanger sequencing to confirm the priming and polymorphism of that InDel marker (**Supplementary File 1**).

### 2.5 Genetic statistics and analysis

Expected heterozygosity (He), observed heterozygosity (Ho) and fixation index (*Fst*) for 10 InDels were calculated in GenAlEx (Peakall and Smouse, 2012). Polymorphic information content (PIC) for 10 InDels was calculated with the ‘pic_calc’ function in Rstudio (version 2022.2.3.492) using package ‘PopGenUtils (R Studio, 2022).’ A distance matrix, calculated using Nei’s genetic distance, was used to create a neighbor-joining (NJ) tree with the ‘bionjs’ algorithm in Rstudio using the ‘ape’ package (Paradis and Schliep, 2019). The NJ tree was visualized using MEGA 11 (Tamura et al., 2021). Population structure was evaluated in STRUCTURE using the admixture, sampling locations, and correlated allele frequencies model with a burn-in of 100,000 and 10,000 MCMC repetitions (Pritchard et al., 2000). The best *k* was determined using the Evanno method (Evanno et al., 2005) *via* Structure Harvester (Earl and vonHoldt, 2012). The best Q-plot, determined using CluMPaK, was visualized using the R package ‘StuctuRly’ (Criscuolo and Angelini, 2020). Genetic groupings were further evaluated using Discriminant Analysis of Principal Components (DAPC) in the R package ‘adegenet’ (Jombart, 2008). Comparison of clusters produced by the NJ method and DAPC was performed by analyzing intersection of sets; sets were assigned by visual examination of clusters produced by NJ algorithm, and posterior DAPC group assignments. Set intersections were calculated and visualized using ‘Intervene’ (**Supplementary Table 4**) (Khan and Mathelier, 2017).

## 3 Results

### 3.1 Yield

Total annual yield of individual plants in 2020 and 2021 ranged from 0.1 g/plant/year (‘China Seed’) to 158.5 g/plant/year (‘Miwa’s Garden’) (**Table 1**). Among the named groups of tea plants, those that recorded the highest total annual yield per plant in both 2020 and 2021 were ‘Small Leaf’ and ‘Fairhope’ (**Table 2**). These groups also had the highest variation. Lowest variance of total annual yield per plant in both 2020 and 2021 was observed in ‘Assamica’ (**Table 2**). In 2020, annual yields for ‘Small Leaf,’ ‘Fairhope,’ ‘Georgian,’ ‘Large Leaf,’ ‘Big Leaf,’ and ‘Assamica,’ were not significantly different at α=0.05. ‘Miwa’s Garden’ was not harvested during that year. In 2021, ‘Small Leaf,’ ‘Fairhope,’ and ‘Miwa’s Garden’ were the highest yielding named groups, with mean yields above 50 grams per plant.

**Table 2:** Mean annual yield by named group of tea plants, variance and the number of plants in each group at PSREU in Citra, FL.

Named Group	2020		2021		Number of plants (n)
	Yield (g/plant)	Variance	Yield (g/plant)	Variance	
‘Assamica’	15.21 ab	2.06	15.72 d	1.84	5
‘Big Leaf’	19.31 ab	7.06	36.29 bc	29.21	40
‘China Seed’	11.56 b	4.08	10.83 d	4.13	27
‘Fairhope’	20.72 a	29.38	50.28 ab	77.40	25
‘Georgian’	20.00 a	21.09	18.71 cd	9.05	20
‘Large Leaf’	14.77 ab	4.47	26.39 cd	19.99	40
‘Small Leaf’	20.76 a	17.68	63.90 a	94.11	12
‘Miwa’s Garden’	NA	NA	54.35 ab	83.27	25

Different lowercase letters following the mean values within each column indicate significant difference (p<0.05) by Duncan’s Multiple Range Test for 2020 and 2021. Plants were harvested in July, September, and November of 2020, and April, June, August, and September of 2021. Mean annual yield was determined by averaging the yield for all harvests within a year by named group. NA, Not available.

### 3.2 Leaf and floral morphology

Multiple morphological traits varied between named groups and some varied among the individual plants within a named group (**Figure 1**). When specific traits were considered individually, the 19 plants observed could be grouped into up to four groups (**Table 3**). Leaf characteristics varied by named group. The ‘Large Leaf’ and ‘Big Leaf’ accessions had the longest and widest leaves, while ‘Small Leaf’ had the shortest and narrowest leaves (**Figure 1**). Leaf colors were assessed to be light green, green, greyed-green, greyed-yellow or yellow green. Leaf color varied within named groups, with only ‘Large Leaf’ and ‘Big Leaf’ showing uniform leaf color. Leaf margins were largely serrulate or biserrate, with ‘Big Leaf’ showing denticulate margins. Leaf pose was moderately upright at between 40 to 75° relative to the stem. Leaf pose was above 57° for all individuals except ‘China Seed 1,’ (40°), ‘Small Leaf 1’ (53°) and ‘Small Leaf 2’ (53°) (**Table 3**). The relative height of pistil and stamen varied among and within the named groups, with only ‘Small Leaf’ individuals showing a uniform characteristic of equal height between pistil and stamen. The style splitting pattern was more uniform within named groups, with ‘Big Leaf’ and ‘Large Leaf’ having the style separating at the base and ascending freely, while ‘Small Leaf,’ ‘Miwa’s Garden,’ and ‘Georgian,’ had a united style that split near the top. Both ‘China Seed’ individuals showed a style that split at the middle of its length. ‘Fairhope’ mostly shared this united style, though one individual, ‘Fairhope 1,’ showed a style that split around the middle of its length (**Supplementary Figure 1**).

**Table 3:** Morphological characteristics from IPGRI descriptor tool for 19 individual plants belonging to 8 named groups of tea.

Individual	Internode Length (mm)	Mature Leaf Color	Leaf Margin	Leaf Length (mm)	Leaf Width (mm)	Leaf Length: Width Ratio	Leaf Pose (°)	Relative height between pistil and stamen	Style splitting
Assamica 1	12.9 ± 2.1 cdefg	2	3	79.0 ± 3.8 bcde	30.2 ± 1.2 defg	2.6	57 ± 4.6 de	3	3
Assamica 2	8.0 ± 2.0 fg	1	3	77.0 ± 7.0 bcdef	32.9 ± 2.3 cd	2.3	66.4 ± 1.6 abcd	1	1
BigLeaf 1	8.7 ± 1.6 efg	2	5	88.2 ± 2.2 ab	42.5 ± 1.4 a	2.1	68.0 ± 3.4 abcd	1	1
BigLeaf 2	7.3 ± 1.1 fg	2	5	71.1 ± 6.3 defg	32.3 ± 3.2 de	2.2	69 ± 1.9 abc	3	1
ChinaSeed 1	10.0 ± 6.3 fg	5	3	71.5 ± 4.7 def	29.9 ± 2.3 defg	2.4	40.0 ± 3.9 f	2	2
ChinaSeed 2	27.3 ± 4 a	1	3	95.4 ± 3.4 a	38.5 ± 1.6 ab	2.5	61 ± 2.4 bcde	1	2
Fairhope 1	11.8 ± 1.6 defg	5	3	51.9 ± 2.1 i	18.1 ± 0.7 j	2.9	59.6 ± 2.4 bcde	2	2
Fairhope 2	18.8 ± 3.2 bc	1	3	64.9 ± 2.1 fgh	30.3 ± 0.9 defg	2.1	75 ± 2.7 a	1	3
Fairhope 3	12.8 ± 1.8 cdefg	2	4	74.7 ± 4.9 cdef	29.5 ± 1.7 defg	2.5	61.0 ± 1.9 bcde	3	3
Georgian 1	15.6 ± 1.9 bcde	5	3	56.7 ± 2.2 hi	22 ± 0.9 hij	2.6	71 ± 1.3 ab	1	3
Georgian 2	7.0 ± 0.7 fg	1	3	69.9 ± 4.9 efg	25.3 ± 2.0 ghi	2.8	60.0 ± 4.2 bcde	3	3
LargeLeaf 1	6.4 ± 0.6 g	2	3	65.6 ± 2.8 fgh	30.8 ± 2.0 def	2.1	69.4 ± 8.3 abc	1	1
LargeLeaf 2	20.0 ± 3.5 b	2	3.5	85.5 ± 4.4 abc	37.6 ± 1.8 bc	2.3	59.0 ± 1.9 cde	3	1
MiwasGarden 1	9.4 ± 1.1 efg	5	3	83.2 ± 2.1 bcd	28.7 ± 1.1 defg	2.9	57 ± 2.5 de	3	3
MiwasGarden 2	14.2 ± 2.4 bcdef	2	3.5	83.1 ± 3.2 bcd	33.6 ± 0.6 cd	2.5	68.0 ± 2.5 abcd	3	3
MiwasGarden 3	11.4 ± 1.2 defg	2	3	65.8 ± 3.5 fgh	26.6 ± 1.1 fgh	2.5	61 ± 2.9 bcde	2	3
SmallLeaf 1	18.2 ± 1.5 bcd	2	3	64.2 ± 2.5 fgh	27.1 ± 0.8 efgh	2.4	53 ± 2.5 e	2	3
SmallLeaf 2	18.9 ± 2.0 bc	5	3	58.8 ± 3.7 ghi	26.7 ± 1.6 fgh	2.2	53.0 ± 4.6 e	2	3
SmallLeaf 3	11.2 ± 1.5 defg	2	3	50.3 ± 3.0 i	21.2 ± 1.5 ij	2.4	70 ± 2.2 abc	2	3

Individuals within each named group are ordered from highest to lowest 2021 yield. Mature leaf color (1= Light green, 2 = Green, 3 = Greyed-green, 4 = Greyed-yellow, 5 = Yellow green). Leaf margin (1 = Entire, 2 = Wavy, 3 = Serrulate, 4 = Biserrate, 5 = Denticulate). Relative height between pistil and stamen (1 = same height, 2 = stamen higher than pistil, 3 = pistil higher than stamen). Splitting of style (1 = geniculate, free for greater part of their length, 2 = ascending, free for about half their length, 3 = united for greater part of the length, the free part short, more or less horizontal and terminal). Letters within a column indicate significantly different (p<0.05) groups identified by Duncan’s multiple range test.

### 3.3 Anthesis characterization

**Figure 2:**
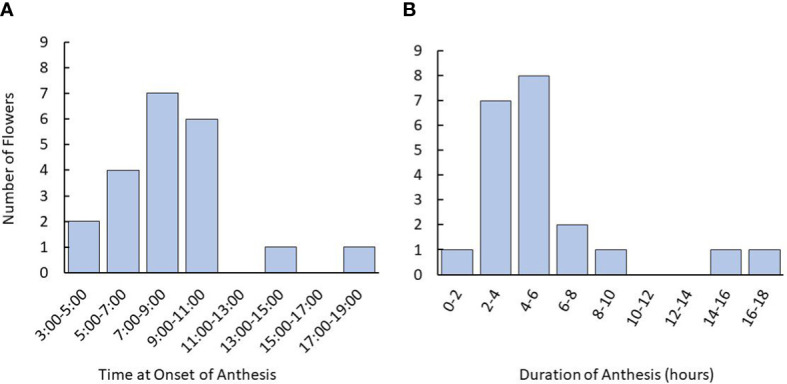
Time and duration of anthesis onset in tea flowers starting from the balloon stage using time lapse photography. **(A)** Time for onset of anthesis of tea flowers observed in flowers. **(B)** Distribution of anthesis duration recorded. Plants used were 3-5 years old. Named groups included were ‘Assamica,’ ‘Black Sea Tea,’ ‘Fairhope,’ ‘Georgian,’ and ‘Small Leaf’. The number of plants used was n=12. The number of flowers observed was n=21.

Timelapse photography revealed that tea flowers largely began anthesis in the early- to mid-morning, typically commencing between 5:00 and 11:00 am (**Figure 2**). Fewer than 10% of flowers began anthesis in the late afternoon or evening. Duration of anthesis was typically within 8 hours of the onset. When anthesis took more than 12 hours, it typically began in the afternoon and paused overnight.

### 3.4 Genetic markers

Analysis of 10 InDel markers across 62 individuals revealed a total of 96 unique alleles (**Table 4**). Fragment sizes ranged from 138 to 358 base pairs. Three loci, CsInDel11, CsInDel17, and CsInDel38, showed notably lower observed heterozygosity (Ho) compared to expected heterozygosity (He). These loci were largely homozygous at the individual level but showed a high degree of variation among individuals. The polymorphism information content (PIC) was similar to the He for all loci. All PIC values were above 0.45, and 7 of 10 InDels showed PIC values at or above 0.50, indicating the InDel markers are highly informative (Botstein et al., 1980). The mean PIC value was 0.67. The fixation index (*Fst*) was measured between the US individuals as one population (n=32) and the Chinese individuals as a second population (n=30). At all loci, *Fst* was <0.05, indicating that there is not sufficient genetic structure to consider the US and Chinese individuals as separate populations.

**Table 4:** Details concerning the apparent number of alleles (Na), range of fragment sizes in base pairs (bp), and observed and expected heterozygosity (Ho and He, respectively) at 10 InDel loci for 62 individual samples of tea and the apparent number of alleles.

Locus	Na	Fragment Size (bp)	He	Ho	PIC	Fst
CsInDel04	9	213-236	0.80 ± 0.02	0.82 ± 0.06	0.80	0.024
CsInDel09	9	203-244	0.71 ± 0.00	0.69 ± 0.09	0.70	0.031
CsInDel11	10	286-322	0.83 ± 0.00	0.38 ± 0.05	0.84	0.035
CsInDel17	20	307-358	0.89 ± 0.00	0.35 ± 0.11	0.90	0.020
CsInDel18	12	283-318	0.74 ± 0.01	0.76 ± 0.15	0.72	0.011
CsInDel19	7	176-207	0.52 ± 0.01	0.32 ± 0.10	0.49	0.009
CsInDel20	4	282-292	0.55 ± 0.03	0.62 ± 0.10	0.46	0.005
CsInDel28	7	216-233	0.54 ± 0.02	0.38 ± 0.00	0.50	0.007
CsInDel38	14	138-350	0.82 ± 0.05	0.43 ± 0.12	0.84	0.034
CsInDel43	4	221-237	0.55 ± 0.00	0.42 ± 0.07	0.47	0.007
10 InDels: US Individuals	73	NA	0.701 ± 0.04	0.562 ± 0.08	NA	NA
10 InDels: Chinese Individuals	82	NA	0.695 ± 0.05	0.483 ± 0.05	NA	NA
10 InDels: US and Chinese Individuals	96	NA	0.712 ± 0.05	0.522 ± 0.06	0.67	NA

Loci where the Ho was less than the He by >0.25 are indicated in bold. Ho and He were calculated for all loci in the entire study population (n=62) and two subpopulations: US Individuals (n=32), and Chinese Individuals (n=30). Polymorphism information content (PIC) was calculated at 10 InDel loci for the entire population (n=62) and a mean PIC value for all loci was also calculated. Fixation index (Fst) was calculated for each locus as a comparison between the two subpopulations. NA, Not applicable.

### 3.5 Genetic diversity and population structure analysis

#### 3.5.1 Genetic distance

**Figure 3:**
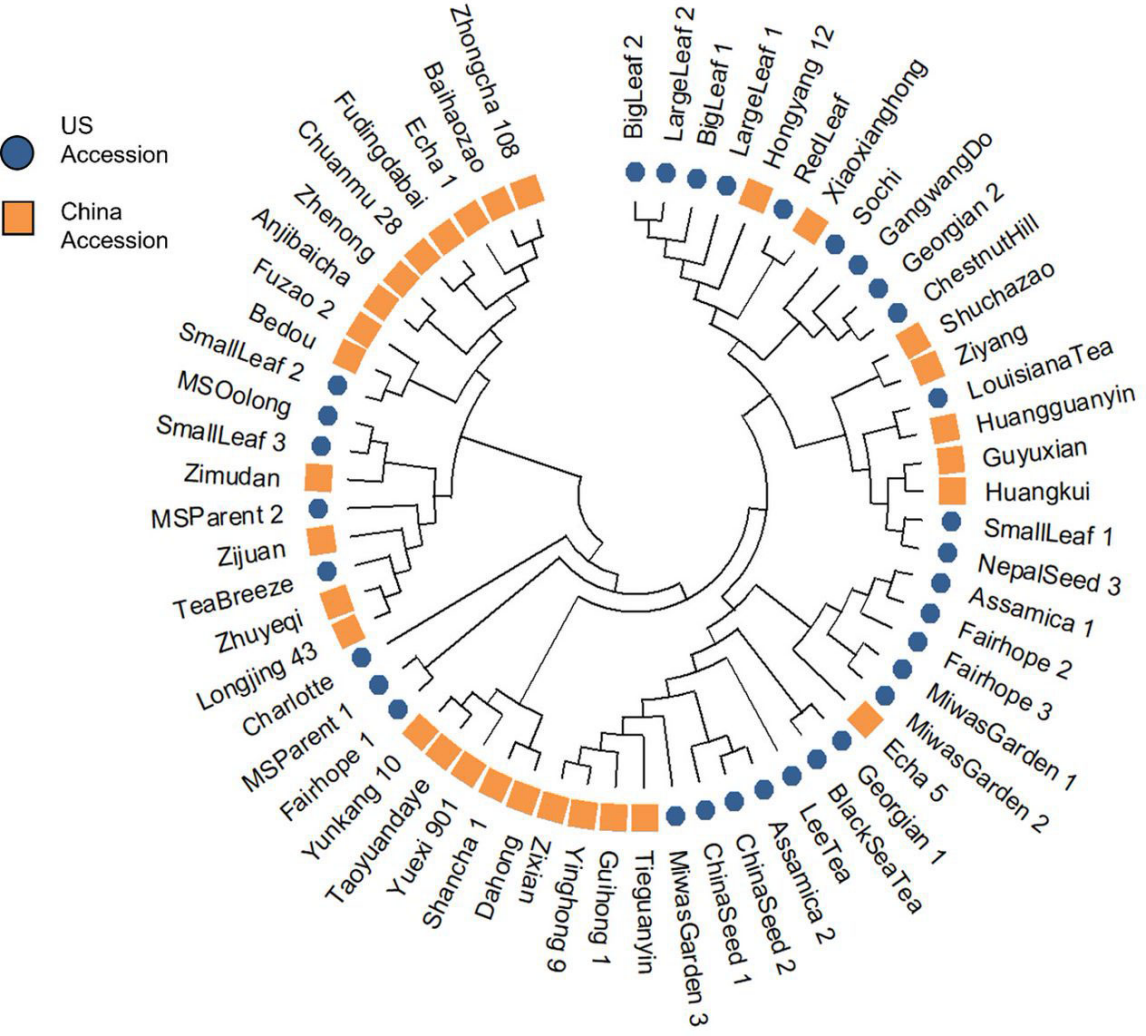
Cluster analysis using Nei’s genetic distance and neighbor-joining algorithm revealed four groups, with a notable separation between CSS reference genome variety ‘Shuchazao’ and CSA reference genome variety ‘Yunkang 10.

The NJ tree revealed four distinct clusters (**Figure 3**; **Supplementary Table 4**). ‘Shuchazao’ and ‘Yunkang 10,’ the reference genome varieties for CSS and CSA respectively, clustered on separate clades. The Chinese variety groupings showed some homology with their previously published groupings according to Liu et al. (2019), notably in the proximity among ‘Shuchazao,’ ‘Guyuxian,’ Ziyan,’ and ‘Xiaoxianghong’ in one clade, ‘Yunkang 10,’ ‘Dahong,’ and ‘Shancha 1’ in another, and ‘Anjibaicha,’ ‘Baihaizhao,’ ‘Chuanmu 28,’ and ‘Fudingdabai’ in a third. Geographical origin of the Chinese varieties was not strongly indicated in the tree. While varieties listed from northern and eastern China (an area encompassing Anhui, Shanxi, and Zhejiang provinces) sometimes occurred close to one another at the tips, there was no overall topology suggesting a strong geographical correlation. Among US tea, several of the seed lot-derived individuals showed considerable distance within named groups, with one ‘Fairhope’ clustering closer to ‘Yunkang 10’ and two others grouping near ‘Shuchazao’ (**Figure 3**). Both ‘China Seed’ individuals grouped next to one another, indicating they were derived from a narrow gene pool. ‘Big Leaf’ and ‘Large Leaf’ individuals had close genetic distance, indicating that these named groups may be derived from the same or very similar original plants. ‘Small Leaf’ individuals showed a broader distance, indicating more genetic diversity within the named group. Notably, ‘Small Leaf 3’ and ‘MS Oolong,’ which are purported to come from the same population (Jason MacDonald, personal communication, October 3, 2021), grouped on the same node. ‘Sochi,’ ‘Gangwang-do,’ ‘Georgian 2’ and ‘Chestnut Hill,’ all of which are named groups known to come from cold regions, grouped together in one node. ‘Black Sea Tea,’ another cold region named group, clustered with ‘Georgian 1’ on a separate node (**Figure 3**).

#### 3.5.2 Population structure

**Figure 4:**
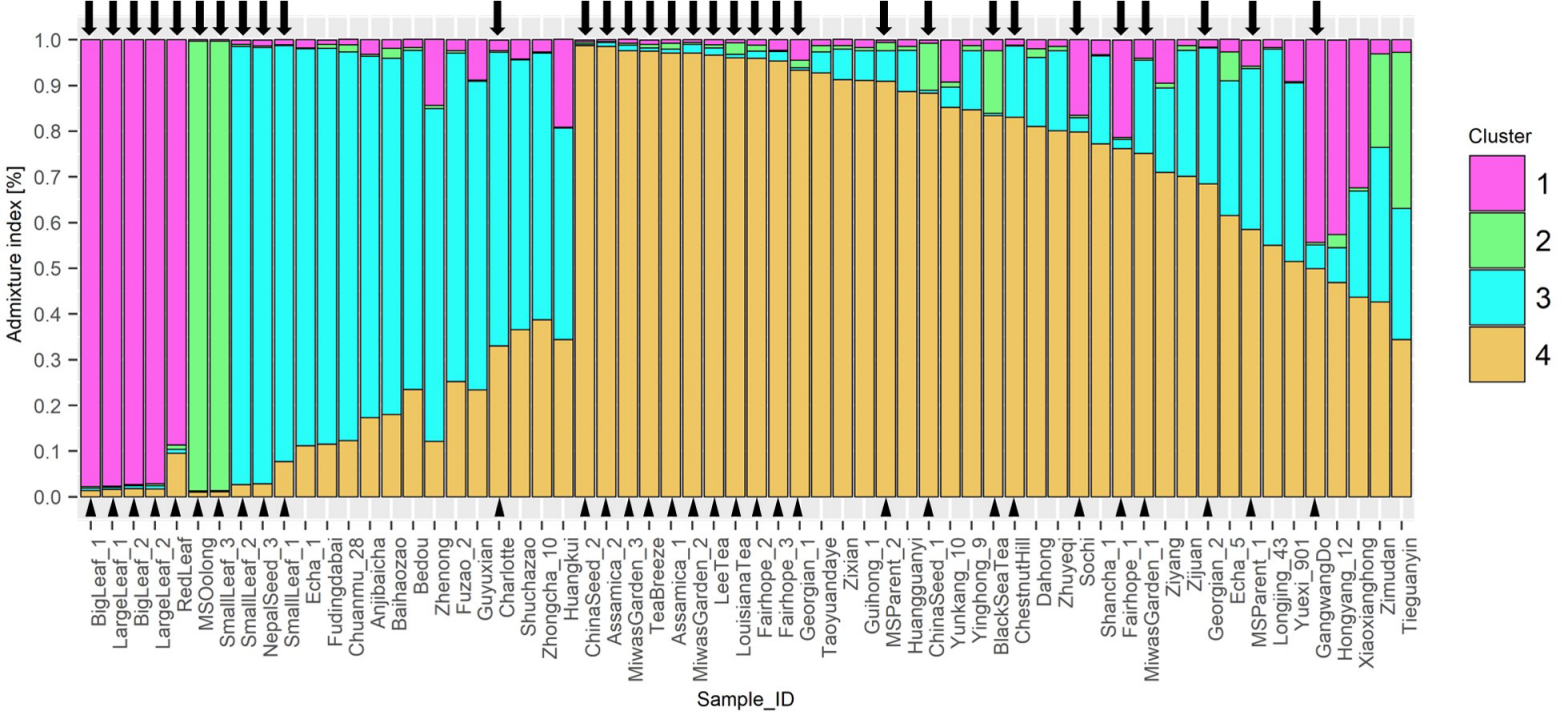
STRUCTURE Q-plot indicating proportion of genome derived from four different ancestral populations based on 10 InDel markers. Analysis of *k*-means indicated 4 ancestral populations using the ΔK method after Evanno et al. (2005). Tea individuals sourced in the US are denoted with a triangle next to the name and an arrow above the corresponding bar.

STRUCTURE analysis indicated 4 ancestral populations (**Figure 4**). Each method included in Evanno’s determination of best *k* indicated k=4. Proximity between individuals seen in the NJ tree are supported by this analysis. ‘Big Leaf’ and ‘Large Leaf’ derive most of their ancestry from a single source, Group 1. ‘Small Leaf 3’ and ‘MS Oolong’ appear to share most of their ancestry from a second source, Group 2. Among the Chinese background population, ‘Tieguanyin’ and ‘Zimudan,’ both from Fujian province, show the highest proportion of ancestry from this group.

Seed lot-derived named groups ‘Fairhope,’ and ‘Georgian’ vary in their ancestral proportions more than ‘Small Leaf,’ also a seed lot-derived named group. ‘Baihaozhao,’ ‘Echa 1,’ ‘Fudingdabai,’ ‘Chuanmu 28,’ ‘Zhenong,’ ‘Anjibaicha,’ ‘Fuzao 2,’ and ‘Bedou,’ which appear together in one node on the NJ tree, are shown in series on the Q-plot with continuous decrease in ancestry from cluster 3 and increasing ancestry from cluster 4 (**Figure 4**). Among these varieties, all except ‘Anjibaicha’ and ‘Fudingdabai’ originate in northeast China. Proximal to this group are ‘Shuchazao,’ ‘Guyuxian,’ and ‘Zhenong 108,’ also from northeast China. ‘Yunkang 10’ shows 85% of its genome from cluster 4, while ‘Shuchazao’ displays more admixture, with 59% of its genome from cluster 3 and 36% from cluster 4. The most admixed individuals are ‘Tieguanyin’ and ‘Zimudan,’ which share a geographical provenance of Fujian province. Among the Chinese varieties, this population structure analysis reflects geographical provenance more strongly than the clusters identified using the NJ method, although both methods used the same set of multilocus genotype data. Varieties originating from northeast China have a higher proportion of ancestry from cluster 3, while those from southwest China largely show a higher proportion of ancestry from cluster 4. ‘Yunkang 10’ and ‘Yinghong 9,’ both CSA varieties, show nearly the same proportion of ancestry from cluster 4 and cluster 2, while ‘Yinghong 9’ shows proportionally more ancestry from cluster 3 (**Figure 4**).

#### 3.5.3 Discriminant analysis of principal components

**Figure 5:**
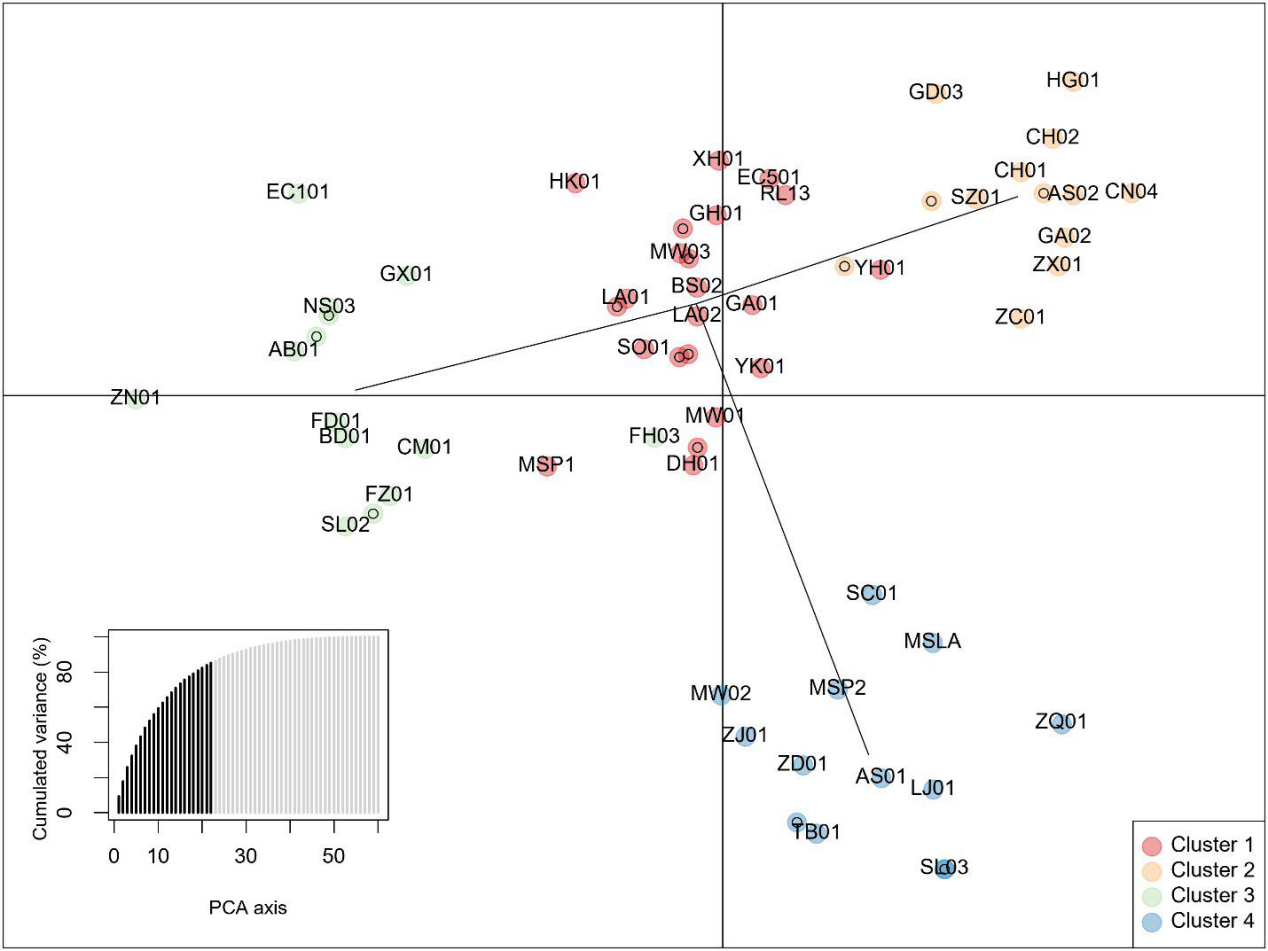
Discriminant analysis of Principal Components of data from 10 InDel markers showing four clusters. The lower left inset shows cumulative variance from 22 retained principal components (PCs) indicated by darker shading. Individuals are identified by 2-letter codes listed in **Supplementary Table 1**. Numbers following the 2-letter codes identify individuals listed in **Table 1**.

Principal components analysis anterior to DAPC indicated 4 groups based on the Bayesian information criterion (BIC) (**Figure 5**). Posterior assignments indicate cluster 1 is the largest with 23 individuals, or 37% of the study population. ‘Yunkang 10’ and ‘Yinghong 9,’ both CSA varieties, appear in Cluster 1. Cluster 3 includes ‘Anjibaicha,’ ‘Baihaozhao,’ ‘Bedou,’ ‘Chuanmu 28,’ ‘Echa 1,’ ‘Fudingdabai,’ ‘Fuzao 2,’ ‘Zhenong,’ and ‘Guyuxiang.’ As previously stated, most of these varieties come from northeast China. This group shows homology with the NJ tree node containing these varieties. ‘Small Leaf 3’ is again associated closely with ‘MS Oolong,’ appearing at the same coordinates on the graph. Accessions from ‘Large Leaf’ and ‘Big Leaf’ appear closely grouped in cluster 1. Individuals from seed lot-derived named groups ‘Fairhope,’ ‘Georgian,’ and ‘Miwa’s Garden’ are split between the clusters, while ‘China Seed’ individuals appear in cluster 2 and ‘Small Leaf’ individuals appear in cluster 4 (**Figure 5**; **Supplementary Table 4**).

#### 3.5.4 Comparison of clusters

**Figure 6:**
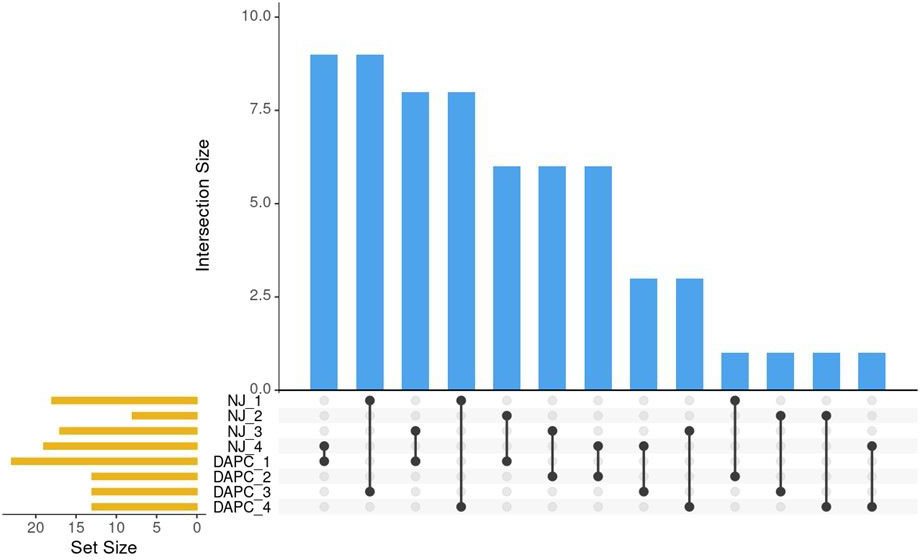
Pairwise comparisons of sets identified by NJ clustering algorithm and by DAPC. Blue bars indicate the number of intersections between sets. Black lines show which sets share these intersections. Orange bars show the number of individuals in the set.

Clusters identified by the Neighbor-joining method (**Figure 3**) and by DAPC (**Figure 5**) were compared using Intersection of Sets (**Figure 6**; **Supplementary Table 4**). The groups identified by the two different methods did not show high rates of intersection, suggesting that the two clustering methods identified different groupings. ‘NJ 2’ is the smallest set with only 8 accessions, 6 of which appear in ‘DAPC 1.’ The next highest rates of intersection were between ‘DAPC 3’ and ‘NJ 1,’ where ‘DAPC 3’ comprises 69% of accessions shared with ‘NJ 1’; and ‘DAPC 4’ and ‘NJ 1,’ where ‘DAPC 4’ comprises 61% of accessions shared with ‘NJ 1’. ‘NJ 1’ is largely split between ‘DAPC 3’ and ‘DAPC 4,’ while the remaining groups show broader dispersal of intersections (**Figure 6**).

## 4 Discussion

The plants included in this study are derived from clonal propagation, selected cuttings from seed-derived plants, or from seed lots (Orrock et al., 2017). Levels of genetic diversity within a named group varied according to propagation method, with clonal varieties ‘Large Leaf’ and ‘Big Leaf,’ suspected to be highly similar germplasm under different trade names, clustering tightly together in the analysis of genetic distance and both analyses of population structure. Individuals from ‘Big Leaf’ and ‘Large Leaf’ were, however, differentiated at two loci. At CsInDel09, ‘Big Leaf 1’ and ‘Large Leaf 1’ had an allele of 212 bp while ‘Big Leaf 2’ and ‘Large Leaf 2’ had an allele of 221 bp. At CsInDel11, ‘Large Leaf 2’ was homozygous for an allele of 301 bp while the other individuals were heterozygous with alleles of 298/301 bp (**Supplementary File 2**). This indicates that these named groups may derive from several selections of a closely related population, instead of purely clonal propagation from a single individual. Congruent to this, the leaf margin trait significantly differed between ‘Large Leaf’ and ‘Big Leaf’ (**Table 3**). The named group ‘Large Leaf’ was reported to be tetraploid (Hembree et al., 2019), and as such the tetraploids may be incompatible with the diploid accessions. The field-tested and greenhouse individuals from both ‘Big Leaf’ and ‘Large Leaf’ show low rates of seed development and maturation, usually aborting the seed by about four months (data not shown).

Though it is a woody perennial crop, tea is not cultivated for a mast of fruit or nuts, but rather for vegetative growth. For this crop, yield and biomass accumulation are closely related parameters (Orrock et al., 2021), with higher yields also indicating that an individual is better adapted to local environmental and climatic conditions. Given the wide array of factors that influence commercial yields, it is difficult to define a “typical yield” for a tea plant, and yield data from varietal garden plots not managed for commercial production may not be predictive of yields for the same germplasm under different conditions. Nevertheless, yield comparisons are necessary to fully investigate poorly described germplasm and make breeding decisions regarding controlled crosses.

The 19 field-grown individuals used in this study were selected based on yield, and all three analyses were able to distinguish at least some high- versus low-yielding genotypes. Cluster analysis of these field-grown accessions may indicate genetic variations related to adaptability in Florida conditions. Among the low-performing named groups, ‘Georgian’ and ‘China Seed,’ occurrence of a few high-yielding individuals may indicate genetic attributes that enable them to perform well despite the majority of the named group showing poor adaptability to the region. They warrant further investigation to preserve advantageous genetic traits that may be present in these individuals and missing from others in the named group. All four individuals representing ‘Big Leaf’ and ‘Large Leaf’ grouped together, which is unsurprising given the other results presented in this study. Since individuals within these named groups show low genetic diversity, poor performance from low yielding ‘Big Leaf’ and ‘Large Leaf’ individuals can possibly be explained by competition, micro-environmental conditions, or patchy distribution of pests and diseases within the field. In the NJ tree of the full 62-member population, the high- and median-yielding ‘Fairhope’ and ‘Miwa’s Garden’ individuals clustered together. The low-yielding individuals from ‘Fairhope’ and ‘Miwa’s Garden’ appeared in a distant cluster from the other individuals in their named group. This division was preserved for ‘Fairhope’ in the population structure analysis, and for ‘Miwa’s Garden’ in the DAPC.

Seed lot-derived named groups ‘Fairhope,’ ‘Small Leaf,’ and ‘Miwa’s Garden’ showed higher genetic diversity within the groups, as well as higher variance in yields. As a self-incompatible species with long-term allogamy, the tea genome shows high heterozygosity and diversity (Chen et al., 2012). Even within a seed lot-derived named group, high genetic diversity is reflected in the cladistic analysis. However, having prior knowledge of the named groups being derived from either seed lot or clonal propagation is not sufficient to predict genetic distance, since some seed lot-derived named groups may have more closely related progenitors, as seen in ‘China Seed’. Cladistic analysis is therefore ideal for estimating genetic distance, both between and within seed lot-derived named groups.

The cluster analysis using the NJ tree may be used to direct choices in breeding, especially of controlled crosses. In an outcrossing organism, inbreeding depression is mitigated by self-incompatibility. Individuals that are closely related may fail to produce viable offspring, or, if offspring survive past the juvenile stage, they may be weak and underperforming. Seed lot-derived individuals from ‘China Seed’ do not show the high variance of yield seen in other seed lot-derived named groups ‘Fairhope’ and ‘Small Leaf.’ Both individuals from ‘China Seed’ clustered nearby each other on the cladistic analysis of genetic distance, indicating low genetic distance, or high genetic similarity, between those individuals. This suggests that ‘China Seed’ has lower genetic variability compared to ‘Fairhope’ and ‘Small Leaf.’ ‘Fairhope,’ ‘Small Leaf,’ ‘Miwa’s Garden,’ and ‘Georgian,’ all seed lot-derived named groups, showed more genetic dissimilarity between individuals included in the study, suggesting these named groups will show more diversity in field production settings and could be useful to identify and select superior performers.

The dendrogram clusters are supported by the population structure results, which estimates four ancestral populations. Long-lived perennials tend to show weak population structure, even among distinct geographical populations; indeed, the likelihood that all individuals studied ultimately belong to a single population was supported by the low *Fst* scores for each InDel locus between US individuals and the Chinese background population (**Table 4**). Therefore, the STRUCTURE analysis was performed with sampling locations included using the LOCPRIOR function (Miller and Gross, 2011). The results for the Chinese background population indicate a different ancestral population for accessions sourced from southwest China compared to those sourced from northeast China. Also separated in this analysis are ‘Shuchazao,’ and ‘Yunkang 10,’ the reference genome specimens for *C. sinensis* var. *sinensis* and *C. sinensis* var. *assamica*, respectively. The population structure analysis suggests that 26 of the 32 US genotypes tested share ancestry with Chinese varieties, a conclusion that is consistent with the historical record of anthropogenically mediated tea movement from East to West. The population structure analysis also shows two subpopulations poorly represented in the Chinese background population but present in the US domestic tea germplasm.

In the population structure analysis, ‘Big Leaf,’ ‘Large Leaf,’ and ‘Red Leaf’ individuals showed a high proportion of ancestry from Group 1. Among the Chinese individuals ‘Hongyang 12’ and ‘Xiaoxianghong’ showed the highest proportion from Group 1. These seven individuals also clustered together in the NJ tree and DAPC. The geographical origins of ‘Big Leaf’ and ‘Large Leaf’ are unconfirmed, but they are thought to derive from a US Department of Agriculture introduction made around 1970 (Jason MacDonald, personal communication, September 8, 2022). ‘Red Leaf,’ is distinguished morphologically by the red color of new shoots and pink flowers (**Supplementary Figure 1**). It has been reported to exist in Japan as far back as 1895 (Makino, 1905; Kitamura, 1950). Kitamura (1950) also notes the large leaf size of red-leaf tea, a trait shared by ‘Big Leaf’ and ‘Large Leaf.’ The historical and genetic evidence suggest that ‘Big Leaf,’ ‘Large Leaf,’ and ‘Red Leaf’ share ancestry from a Japanese population of tea. The genetic evidence further suggests that the progenitors of this population contributed some genetic material to Chinese individuals ‘Hongyang 12,’ ‘Xiaxianghong,’ and Korean individual ‘Gangwang-do’ (**Figure 3**).

‘Small Leaf 3’ and ‘MS Oolong’ showed much of their ancestry from Group 2, another group that is not strongly represented in the Chinese background population. The University of Florida ‘Small Leaf’ named group came from the same nursery stock as the ‘MS Oolong’ individual. This named group is thought to originate from clonal propagules sourced from Charles Shepard’s Pinehurst tea plantation in South Carolina (Jason McDonald, personal communication, September 8, 2022). There is a stark division in ancestry between ‘MS Oolong’ and ‘Small Leaf 3’ compared to ‘Small Leaf 1’ and Small Leaf 2,’ the latter of which share more ancestry with individuals from northeast China (**Figure 3**) and are morphologically comparable (**Table 3**). The genetic evidence suggests a number of cuttings were taken from genetically dissimilar plants and sold under a single trade name; a conjecture consistent with the historical record showing tea from several different regions were planted at Pinehurst (Walcott, 1999). The putative origin of ‘MS Oolong’ and ‘Small Leaf 3’ is more difficult to ascertain. Of the recorded plants at Pinehurst, an Assam hybrid seems unlikely because of the small leaf size. A Chinese variety also seems unlikely, since the ancestral Group 2 would be better represented in the genetic structure analysis of the Chinese population. These individuals may have therefore originated from a Japanese tea population that was discrete from the ‘Big Leaf,’ ‘Large Leaf,’ and ‘Red Leaf’ progenitors. Though the genetic diversity among commercial Japanese tea varieties is low compared to other tea-growing regions (Ni et al., 2008), this is due to the high density of ‘Yabukita’ plantings and its heavy use as a breeding parent. ‘Yabukita’ was not selected until the early 20^th^ century, about 50 years after the establishment of Pinehurst tea plantation. Japanese tea varieties from the Shizuoka and Uji regions, the two main sites of tea’s introduction into Japan from China around the 13^th^ century, were likely sourced from distinct populations within China (Yamashita et al., 2019). Additionally, recent genetic analyses using SNP markers have shown these varieties share little genetic similarity with extant Chinese varieties (Yamashita et al., 2019). However, further studies involving genetic comparison between Japanese tea varieties and US-sourced tea accessions are necessary to confirm this hypothesis.

DAPC, by minimizing variation within groups, can be useful for balancing breeding decisions based on NJ or similar algorithms, which are designed to show genetic distance. DAPC is used in this study instead of another commonly used measure of genetic variation, the PCA. DAPC does in fact begin by transforming the data using PCA, which allows for an informed selection of the number of groups using the BIC to determine the best *k.* While PCA helps to interpret total genetic variation, DAPC is more suited for analyzing variance among groups, while minimizing within-group variation. (Jombart and Collins, 2015). DAPC is not based on a distance matrix like the NJ algorithm, but rather on allele frequencies. Reviewing the results using intersection of sets provides insight into the utility of the different clustering analyses. The DAPC posterior assignment groupings showed homology with node tip assignments seen in the NJ clusters, and ancestry estimates produced by the population structure analysis. However, group assignments differed between the DAPC and NJ algorithm, to some extent; this divergence is explored in the Intersection of Sets analysis (**Figure 6**). Notable shared assignments between the DAPC and NJ method included named groups shown to be closely related such as ‘Big Leaf’ and ‘Large Leaf.’ Since DAPC is designed to minimize variation within groups, some of the seed lot-derived named groups appeared in the same cluster, while on the NJ tree, they appeared in different clades. The DAPC results are in keeping with the population structure analysis, which shows ‘Small Leaf’ and ‘Miwa’s Garden’ in the same DAPC groups, and also having similar proportions of ancestry to one another on the population structure analysis.

The investigation into timing and duration of anthesis presents opportunities to cross pairs that would otherwise be temporally isolated. Finally, it should be noted that the breeding recommendations presented here are based on yield and do not take into account questions of quality. Further studies examining the tea produced from named groups and individuals under Florida field conditions are required to investigate horticultural and genetic effects on quality of Florida-grown tea.

As an allogamous plant with a highly heterozygous genome and broad geographic range of cultivation, the potential for tea germplasm to possess adaptations to specific environmental conditions is high. Inferring regional suitability may be possible using genetic analysis and observations of regional viability. ‘Chestnut Hill’ was selected from Morris Arboretum near Philadelphia, which is USDA Plant Hardiness Zone 7a and experiences winter low temperatures from -17.8 to -15°C (USDA, 2012). This specimen was obtained by the Arboretum in 1953 (Goff, 2021). Given the warming trend of the last 50 years, it has likely survived even colder temperatures. ‘Gangwang-do,’ ‘Sochi,’ and ‘Georgian 2’ clustered together with ‘Chestnut Hill’ on the NJ tree, indicating that they, too, may be suited for relatively colder winter temperatures. This analysis is consistent with the reported origins of these named groups in the colder parts of the tea growing range: mountains near the Korean DMZ, and the region around Sochi, Russia.

The poor yield performance of ‘Assamica’ in Florida field conditions is worth noting, especially because this named group was predicted to be one of the better performers for this area due to CSA being mostly cultivated in the relatively warmer tea growing regions (Wei et al., 2018). Knowing that CSA subspecies available in the US likely originated from the Indian Himalayas, it is possible that the field-tested ‘Assamica’ individuals are either shade-adapted or require higher altitudes in areas reaching above 30°C, if not both (Carr, 2018).

Based on yield, ‘Fairhope’ individuals perform well in Florida conditions, as do ‘Miwa’s Garden,’ ‘Big Leaf’ and ‘Small Leaf.’ The polyploidy of ‘Big Leaf’ and ‘Large Leaf’ is consistent with broader environmental adaptability (Ramsey, 2011). One of the ‘Small Leaf’ accessions, ‘Small Leaf 3,’ has had low yields in Florida, but shows high homology with ‘MS Oolong’ in the population structure analysis, NJ tree, and DAPC. The ‘MS Oolong’ individual is an established plant at a working tea farm in Mississippi and was sourced from the same tea seed farm as the named group ‘Small Leaf.’ The ‘MS Oolong’ plants are productive in an area with cooler summer and winter temperatures than Florida, among other environmental differences. Given the high genetic similarity between these two individuals, and the dichotomy in ancestry within ‘Small Leaf’ demonstrated by the population structure analysis, it is likely that some ‘Small Leaf’ plants are generally more cold-hardy and less likely to be heat tolerant. Indeed, this highlights some possibly rare attributes of the surviving ‘Small Leaf’ plants field-grown in Florida.

## Data availability statement

The original contributions presented in the study are included in the article/**Supplementary Material**. Further inquiries can be directed to the corresponding author.

## Author contributions 

BR and BSR designed the outline of this study and collected the resources needed. CC completed the experimental research, computational data analyses and prepared the manuscript. All authors contributed to the article and approved the submitted version.
