# Supplementary material for: Genetic and morphological characterization of United States tea (Camellia sinensis): insights into crop history, breeding strategies, and regional adaptability
Source: Front Plant Sci. 2023 May 12;14:1149682. doi: 10.3389/fpls.2023.1149682 (PMC10213625; doi:10.3389/fpls.2023.1149682)
Supplement: Supplementary file 1 [file Table_1.docx]

**Supplementary Table 1.** Named group, abbreviation, number of plants in UF collection, propagation method, source, and putative origin of the plants evaluated. The abbreviation is a two-letter code unique to each named group. Sources are Camellia Forest Nursery (CF), Mississippi State University (MSU), Great Mississippi Tea Company (GM), CamToo Nursery (CT), Buddy Lee Nursery (BL), and Fairhope Tea Plantation (FH).

| Named group | Abbreviation | Number of Individuals Included in Genetic Diversity Study | Propagation Method | Source | Putative Origin |
| --- | --- | --- | --- | --- | --- |
| ‘Assamica’ | AS | 2 | Seed | CF | Unknown |
| ‘Big Leaf’ | BL | 2 | Clonal | MSU | Unknown |
| ‘China Seed’ | CH | 2 | Seed | CF | China |
| ‘Fairhope’ | FH | 3 | Seed | FH | Fairhope, AL |
| ‘Georgian’ | GA | 2 | Seed | MSU | Black sea region, Georgia |
| ‘Miwa’s Garden’ | MW | 3 | Seed – Single plant selection | CF | Unknown |
| ‘Large Leaf’ | LA | 2 | Clonal | CF | Unknown |
| ‘Small Leaf’ | SL | 3 | Seed | CF | Japan |
| ‘MS Parent’ | MSP | 2 | Unknown | GM | Unknown |
| ‘MS Oolong’ | MSOC | 1 | Unknown | GM | Japan |
| ‘Louisiana Tea’ | MSLA | 1 | Clonal | BL | Unknown |
| ‘Lee Tea’ | MSLT | 1 | Clonal | BL | Louisiana |
| ‘Black Sea Tea’ | BS | 1 | Seed | CF | Black Sea region, Georgia |
| ‘Charlotte’ | CL | 1 | Unknown | CF | Florida, US |
| ‘Chestnut Hill’ | CH | 1 | Clonal | CF | Morris Arboretum, Philadelphia, PA |
| ‘Gangwang-do’ | GD | 1 | Seed | CF | South Korea DMZ |
| ‘Nepal Seed’ | NS | 1 | Seed | MSU | Nepal |
| ‘Red Leaf’ | RL | 1 | Clonal | CT | Japan |
| ‘Sochi’ | SO | 1 | Seed | CF | Black Sea region, Georgia |
| ‘Teabreeze’ | TB | 1 | Unknown | CF | Unknown |

**Supplementary** **Table 2**. Chinese varieties used as a background population in the genetic diversity analyses, supplied by Chaolin Wei at Anhui Agricultural University (Heifei, China). Included is the accession name, a unique two-letter code, the cultivar status (National, Regional, or Local registry), and region of origin (Liu et al., 2019).

| Chinese Variety | Abbreviation | Cultivar Status | Region of Origin |
| --- | --- | --- | --- |
| Anjibaicha’ | AB | Provincial | Zhejiang |
| ‘Bedou’ | BD | Local | Fujian |
| ‘Baihaozhao’ | BZ | National | Hunan |
| ‘Chuanmu 28’ | CM | Provincial | Sichuan |
| ‘Dahong’ | DH | Unknown | Fujian |
| ‘Echa 1’ | EC1 | National | Hubei |
| ‘Echa 5’ | EC5 | National | Hubei |
| ‘Fudingdabai’ | FD | National | Fujian |
| ‘Fuzao 2’ | FZ | National | Anhui |
| ‘Guihong 1’ | GH | Unknown | Guanxi |
| ‘Guyuxian’ | GX | Local | Anhui |
| ‘Huangguanyin’ | HG | National | Fujian |
| ‘Huangkui’ | HK | Local | Anhui |
| ‘Hongyan 12’ | HY | National | Guangdong |
| ‘Longjing 43’ | LJ | National | Zhejiang |
| ‘Shancha 1’ | SC | Provincial | Shanxi |
| ‘Shuchazao’ | SZ | National | Anhui |
| ‘Taoyuandaye’ | TD | Provincial | Hunan |
| ‘Tieguanyin’ | TY | National | Fujian |
| ‘Xiaoxianghong’ | XH | Provincial | Hunan |
| ‘Yinghong 9’ | YH | Provincial | Guangdong |
| ‘Yunkang 10’ | YK | National | Yunnan |
| ‘Yuexi 901’ | YX | Unknown | Anhui |
| ‘Zhongcha 108’ | ZC | National | Zhejian |
| ‘Zimudan’ | ZD | National | Fujian |
| ‘Zijuan’ | ZJ | Local | Yunnan |
| ‘Zhenong 113’ | ZN | National | Zhejiang |
| ‘Zhuyeqi’ | ZQ | National | Hunan |
| ‘Zixian’ | ZX | Local | Sichuan |
| ‘Ziyan’ | ZY | Local | Sichuan |

**Supplementary Table 3**. Primer sequences and expected product sizes of the 10 InDels (Liu et al., 2019) used to estimate genetic diversity of US tea germplasm.

| **Marker ID** | **Forward Primer (5’ to 3’)** | **Reverse Primer (5’ to 3’)** | **Expected Product Size (bp)** |
| --- | --- | --- | --- |
| CsInDel04 | TAATCTGACGCAGCGCTTTTG | GGACCTTTCAGCTTCTCTCCC | 214-242 |
| CsInDel08 | AGACAACTCCGGGTAATGGA | GGCTTGTGGTTCTTCAGGTA | 206-215 |
| CsInDel09 | GGTTGTGCAGTTTGGGAGTTG | GGTTGAGGTTGAGGTTGAGGT | 201-248 |
| CsInDel11 | GCAGTAGTTGTTGTGGGGAGA | TGGTGGCACTTTGTCTCACTT | 292-332 |
| CsInDel17 | ATTGAGAATGGCGGAAGTGGT | GGGTTGCAGAGTTAAATTCGGG | 306-354 |
| CsInDel18 | GGTTAAGCGTTTGGGTCCTT | ACACACCAACCCTCTCCTTA | 283-326 |
| CsInDel19 | CCCTTCGCATATTCCACACA | TGTCATACCCCAGCATCTTG | 180-214 |
| CsInDel20 | GTAGTGGGTATGTGCTGGTG | ACCGGGTTTCAAATTGCTTT | 285-297 |
| CsInDel28 | AAAATGAAGAGGAGGGCGACA | AGCCGGCTGTACAAGAAATCA | 213-253 |
| CsInDel38 | AGAGACAGAGAGAAGAGCGA | AAATGGAAAAAGACCGGGGT | 109-340 |
| CsInDel43 | GCAGGAGACAGATCAGAACG | TGAAAACGGCCATCAAGAGT | 226-254 |
| CsInDel45 | TCTCTCCAGCCTCATACCAG | CGCTGCAAATAACGACCCTA | 281-321 |

**Supplementary Table 4.** Groups identified by neighbor-joining algorithm (NJ Sets) and Discriminant Analysis of Principal Components (DAPC Sets) were compared using Intersection of Sets analysis to estimate homology between groupings produced by each method.

| **NJ Sets** |  |  |  |
| --- | --- | --- | --- |
| **NJ_1** | **NJ_2** | **NJ_3** | **NJ_4** |
| Anjibaicha | Charlotte | Assamica_1 | BigLeaf_1 |
| Baihaozao | Dahong | Assamica_2 | BigLeaf_2 |
| Bedou | Fairhope_1 | BlackSeaTea | ChestnutHill |
| Chuanmu_28 | MSParent_1 | ChinaSeed_1 | GangwangDo |
| Echa_1 | Shancha_1 | ChinaSeed_2 | Georgian_2 |
| Fudingdabai | Taoyuandaye | Echa_5 | Guyuxian |
| Fuzao_2 | Yuexi_901 | Fairhope_2 | Hongyang_12 |
| Longjing_43 | Yunkang_10 | Fairhope_3 | Huangguanyin |
| MSOolong |  | Georgian_1 | Huangkui |
| MSParent_2 |  | Guihong_1 | LargeLeaf_1 |
| SmallLeaf_2 |  | LeeTea | LargeLeaf_2 |
| SmallLeaf_3 |  | MiwasGarden_1 | LouisianaTea |
| TeaBreeze |  | MiwasGarden_2 | NepalSeed_3 |
| Zhenong |  | MiwasGarden_3 | RedLeaf |
| Zhongcha_108 |  | Tieguanyin | Shuchazao |
| Zhuyeqi |  | Yinghong_9 | SmallLeaf_1 |
| Zijuan |  | Zixian | Sochi |
| Zimudan |  |  | Xiaoxianghong |
|  |  |  | Ziyang |
| **DAPC Sets** |  |  |  |
| **DAPC_1** | **DAPC_2** | **DAPC_3** | **DAPC_4** |
| BigLeaf_1 | Assamica_2 | Anjibaicha | Assamica_1 |
| BigLeaf_2 | ChestnutHill | Baihaozao | Fairhope_2 |
| BlackSeaTea | ChinaSeed_1 | Bedou | Longjing_43 |
| Dahong | ChinaSeed_2 | Charlotte | LouisianaTea |
| Echa_5 | GangwangDo | Chuanmu_28 | MiwasGarden_2 |
| Fairhope_1 | Georgian_2 | Echa_1 | MSOolong |
| Fairhope_3 | Huangguanyin | Fudingdabai | MSParent_2 |
| Georgian_1 | LeeTea | Fuzao_2 | Shancha_1 |
| Guihong_1 | Shuchazao | Guyuxian | SmallLeaf_3 |
| Hongyang_12 | Tieguanyin | NepalSeed_3 | TeaBreeze |
| Huangkui | Zhongcha_108 | SmallLeaf_1 | Zhuyeqi |
| LargeLeaf_1 | Zixian | SmallLeaf_2 | Zijuan |
| LargeLeaf_2 | Ziyang | Zhenong | Zimudan |
| MiwasGarden_1 |  |  |  |
| MiwasGarden_3 |  |  |  |
| MSParent_1 |  |  |  |
| RedLeaf |  |  |  |
| Sochi |  |  |  |
| Taoyuandaye |  |  |  |
| Xiaoxianghong |  |  |  |
| Yinghong_9 |  |  |  |
| Yuexi_901 |  |  |  |
| Yunkang_10 |  |  |  |
