# Supplementary material for: Genetic and morphological characterization of United States tea (Camellia sinensis): insights into crop history, breeding strategies, and regional adaptability
Source: Front Plant Sci. 2023 May 12;14:1149682. doi: 10.3389/fpls.2023.1149682 (PMC10213625; doi:10.3389/fpls.2023.1149682)
Supplement: Supplementary file 3 [file Image_1.pdf]

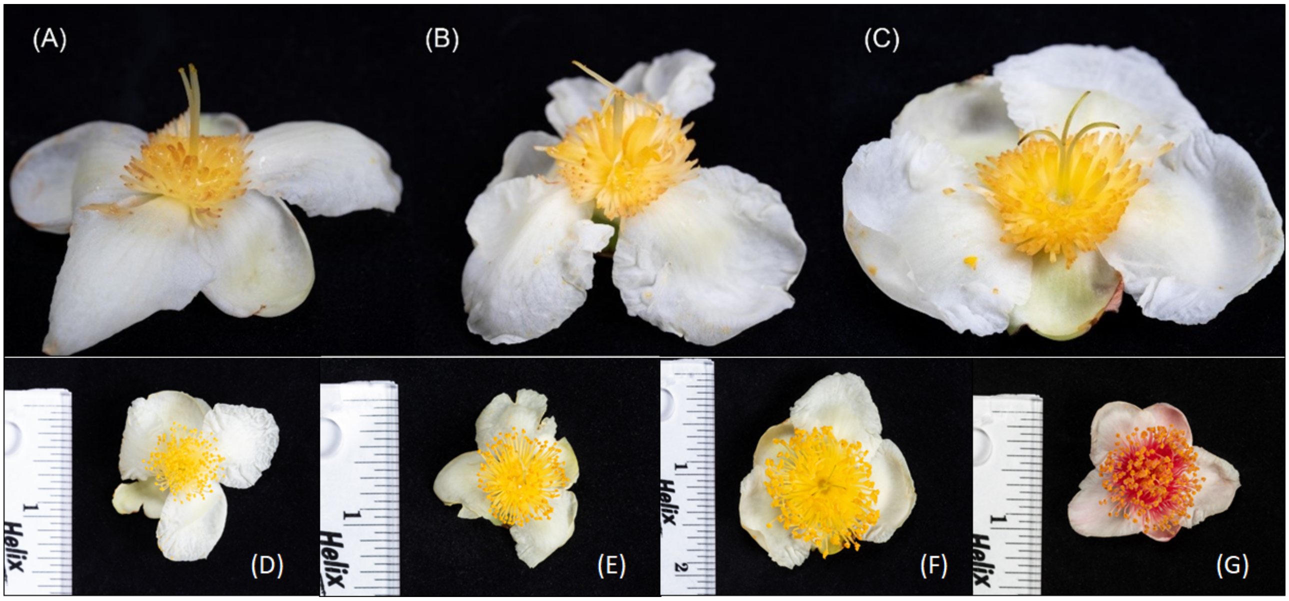

Supplementary Figure 1. Floral morphological characteristics. (A), (B), and (C) show three different types of style splitting, where in (A) the style is united for the greater part of its length, (B) the style splits at about the middle, and (C) the style splits at the base, just above the ovary of flowers from named groups ‘Georgian’, ‘Fairhope’ and ‘Large Leaf’ respectively. The lower panel shows floral size and petal color for (D) ‘Georgian,’ (E) ‘Fairhope’ (F) ‘Large Leaf’ and (G) ‘Red Leaf.’ ‘Large Leaf’ shows a relatively larger floral diameter. ‘Fairhope’ and 'Large Leaf' have a duller white petal color compared to ‘Georgian,' and 'Red Leaf' has pink petals and filaments.
