# Supplementary material for: Genetic and morphological characterization of United States tea (Camellia sinensis): insights into crop history, breeding strategies, and regional adaptability
Source: Front Plant Sci. 2023 May 12;14:1149682. doi: 10.3389/fpls.2023.1149682 (PMC10213625; doi:10.3389/fpls.2023.1149682)
Supplement: Supplementary file 4 [file DataSheet_1.pdf]

Supplementary File 1.

DNA sequences amplified from the genomic DNA of five genotypes of tea using the primer pair CsIndel17. The DNA sequences are single pass sequences from 'Ziyang', 'Teabreeze', 'Assmica2', 'Gangwando', and 'Assamica1' and deletions are shown in dotted lines identified by comparison to tea reference genome .

>1-CsIndel17F Ziyang.ab1
TCTGTNGGTTCCATNGCGNGGCTCATACTTCTTGGCTTCTTTGTCTACATAGAGAC--------------
TCTCTCTCTCTCTCTCATATTTCCGCTTTCAAACCCACAACCATTTTTTTAATATTGCACCATTTT
GCTTTTTGTTTATTCTTCAACTCNCNNGGGATATCTCTTCATTATTTTTATTTTCTTTTATTTCTCTAACTTGGGCTTATTC
GNACTAATGCATATATATATATACATATATATAACTCAATCTCCCGAATANAACT
CTGCAACACA

>2-CsIndel17F Teabreeze.ab1
TCTGTTGGTTCCATTGCGTGGCTCATACTTCTTGGCTTCTTTGTCTACAT--AGAC--------
TCTCTCTCTCTCTCTCTCTCTCTCATATTTCCACTTTCAAACCCACAACCATTTTTTTAATATTTCACCATTTT
CCTTTTTGTTTATTCTTCAACTCACCTGGCATATCTCTTCATTATTTTTATTTTCTTTTATTTCTCTAACTTGGGCTTATTCC
TACTAATAC--ATATATATATACATATATATAACTCAATCTCCCGAATTTAA
CTCTGCAACACA

>3-CsIndel17F Assamica 2.ab1
TCTGTTGGTTCCATTGCGTGGCTCATACTTCTTGGCTTCTTTGTCTACATAGAGAC----
TCTCTCTCTCTCTCTCTCTCTCTCTCTCATATTTCCACTTTCAAACCCACAACCATTTTTTTAATATTTCACCATTT
TCCTTTTTGTTTATTCTTCAACTCACCTGGCATATCTCTTCATTATTTTTATTTTCTTTTATTTCTCTAACTTGGGCTTATTC
CTACTAATAC--ATATATATAT--ATATATATATCTCTCTCTCCCCAAAATN
ACNCNCCCACACA

>6-CsIndel17F Gangwando.ab1
TCTGTTGGTTCCATTGCGTGGCTCATACTTCTTGGCTTCTTTGTCTACAT--
AGACTCTCTCTCTCTCTCTCTCTCTCTCTCTCTCATATTTCCACTTTCAAACCCACAACCTTTTTTTTAATATTTCACCTT
TTTCTTTTTTGTTTATTCTTCAACTCACCTGGTATATCTCTTCATTATTTTTATTTTCTTTTATTTCTCTAACGTGGGCTTAT
TCCTACAAATAC--ATATATATATACATATATATAACTCACTCTCCAAAAT
TAAACNNNGCCACACA

>7-CsIndel17F Assamica 1.ab1
TCTGTTGGTTCCATTGCGTGGCTCATACTTCTTGGCTTCTTTGTCTACATAGAGACTCTCTCTCTCTCTCTCTCTCTCT
CTCTCATATTTCCACTTAAAAACTCACAACTTTTTTTTATATATTTCACCTTT
TTTCTTTTTGTTTATTCTTCCACTCACTTGGTATATCTCTTNATTATTTTTATTTTCTTTTATTTCTCTAACGTGGGCTTATT
CCTACAAATAC--ATATATATATACATATATATAACTCTCTCTCCCAAATTT
NACNCNGCCACACA
